# Obesity: Clinical Impact, Pathophysiology, Complications, and Modern Innovations in Therapeutic Strategies

**DOI:** 10.3390/medicines12030019

**Published:** 2025-07-28

**Authors:** Mohammad Iftekhar Ullah, Sadeka Tamanna

**Affiliations:** 1 Department of Medicine, University of Mississippi Medical Center, 2500 N State St., Jackson, MS 39216, USA; sadeka.tamanna@va.gov; 2 G. V. (Sonny) Montgomery VA Medical Center, 1500 E Woodrow Wilson Ave., Jackson, MS 39216, USA

**Keywords:** obesity, metabolic syndrome, weight management, emerging therapies, pharmacotherapy, GLP-1 receptor agonists, Dual GIP/GLP-1 agonists

## Abstract

Obesity is a growing global health concern with widespread impacts on physical, psychological, and social well-being. Clinically, it is a major driver of type 2 diabetes (T2D), cardiovascular disease (CVD), non-alcoholic fatty liver disease (NAFLD), and cancer, reducing life expectancy by 5–20 years and imposing a staggering economic burden of USD 2 trillion annually (2.8% of global GDP). Despite its significant health and socioeconomic impact, earlier obesity medications, such as fenfluramine, sibutramine, and orlistat, fell short of expectations due to limited effectiveness, serious side effects including valvular heart disease and gastrointestinal issues, and high rates of treatment discontinuation. The advent of glucagon-like peptide-1 (GLP-1) receptor agonists (e.g., semaglutide, tirzepatide) has revolutionized obesity management. These agents demonstrate unprecedented efficacy, achieving 15–25% mean weight loss in clinical trials, alongside reducing major adverse cardiovascular events by 20% and T2D incidence by 72%. Emerging therapies, including oral GLP-1 agonists and triple-receptor agonists (e.g., retatrutide), promise enhanced tolerability and muscle preservation, potentially bridging the efficacy gap with bariatric surgery. However, challenges persist. High costs, supply shortages, and unequal access pose significant barriers to the widespread implementation of obesity treatment, particularly in low-resource settings. Gastrointestinal side effects and long-term safety concerns require close monitoring, while weight regain after medication discontinuation emphasizes the need for ongoing adherence and lifestyle support. This review highlights the transformative potential of incretin-based therapies while advocating for policy reforms to address cost barriers, equitable access, and preventive strategies. Future research must prioritize long-term cardiovascular outcome trials and mitigate emerging risks, such as sarcopenia and joint degeneration. A multidisciplinary approach combining pharmacotherapy, behavioral interventions, and systemic policy changes is critical to curbing the obesity epidemic and its downstream consequences.

## 1. Introduction

Obesity has emerged as one of the most pressing global health crises of the 21st century, with its prevalence nearly tripling since 1975 [1]. Rising obesity rates place enormous strain on healthcare systems, economies, and personal health and lead to millions of avoidable deaths each year from cardiovascular disease, type 2 diabetes, various cancers, and other complications [2]. In the past, obesity management has been a challenge, from ineffective lifestyle interventions to high-risk pharmacotherapies and invasive surgical procedures. Early weight loss medications, such as fenfluramine (Fen-Phen) and sibutramine, were withdrawn due to severe cardiovascular and psychiatric side effects [3,4]. Bariatric surgery, while effective for sustained weight loss, remains inaccessible to many due to cost, procedural risks, and long-term nutritional deficiencies [5]. These limitations underscore the urgent need for safer, more effective therapeutic strategies.

Recent advancements in obesity pharmacotherapy, particularly glucagon-like peptide-1 (GLP-1) receptor agonists (e.g., semaglutide, tirzepatide), revolutionized treatment paradigms, demonstrating unprecedented efficacy in clinical trials [6,7]. Yet, these breakthroughs are not without challenges, including high costs, accessibility barriers, and concerns over long-term safety and weight regain upon discontinuation [8].

This review explores the complex nature of obesity, covering its underlying biology, social and economic consequences, related health conditions, and evolving treatment options. We assess both historical and current therapies, examine how new medications work and their limitations, and consider future strategies for managing obesity. By bringing together the latest evidence, this article offers a clear overview of obesity as a chronic disease that demands coordinated, multidisciplinary approaches.

## 2. Definition and Classification of Obesity

Obesity is a complex, chronic disease marked by abnormal or excessive fat accumulation that adversely impacts health, elevating the risk of metabolic, cardiovascular, and musculoskeletal disorders. Historically, obesity has been defined using body mass index (BMI), a simple metric calculated as weight in kilograms divided by height in meters squared. According to the World Health Organization, adults with a BMI of 30 or higher are classified as obese, with subcategories for Class I (30–34.9), Class II (35–39.9), and Class III (≥40) obesity [9]. While BMI remains widely used due to its simplicity, its limitations have spurred calls for more nuanced diagnostic frameworks.

In recent years, the medical community has shifted toward a multidimensional definition of obesity. A landmark consensus statement has been proposed classifying obesity into *clinical* and *pre-clinical* stages to better capture its heterogeneous nature [9]. Clinical obesity is diagnosed when excess adiposity directly impairs organ function or limits daily activities—for example, causing hypertension, obstructive sleep apnea, or severe joint degeneration. In contrast, pre-clinical obesity applies to individuals with excess fat accumulation but no overt complications, though they remain at elevated risk for future diseases. This framework integrates not only BMI but also waist circumference, biomarkers of metabolic dysfunction, and evidence of obesity-related complications, offering a more holistic assessment.

Ethnic and demographic variations further complicate classification. For instance, Asian populations experience cardiometabolic risks at lower BMI thresholds than Caucasians, prompting region-specific guidelines that define obesity as BMI ≥25 in many Asian countries [10]. Similarly, the Edmonton Obesity Staging System (EOSS) incorporates mental health and quality of life metrics, recognizing that obesity’s impact extends beyond physical health [11]. Despite these advancements, debates persist about how to balance practicality with precision, particularly in resource-limited settings.

## 3. Limitations of Current Definitions

While BMI’s simplicity suits large studies and routine care, it has clear limitations. It cannot distinguish lean muscle mass from fat tissue, leading to the misclassification of athletes or older adults with low muscle mass as overweight or obese [12]. It also overlooks fat distribution patterns; visceral fat, which surrounds internal organs and drives metabolic dysfunction, is a stronger predictor of disease risk than subcutaneous fat but remains invisible to BMI [13]. For example, individuals with normal BMI but high visceral fat are termed as “metabolically obese normal weight”, but their cardiovascular risks are comparable to those with clinical obesity [14].

Ethnic differences further weaken its accuracy. Standard BMI cutoffs miss risk in South Asian groups, who tend to have more visceral fat and insulin resistance at a lower BMI, and overstate risk in Black populations, who often carry more muscle mass [15]. Pediatric obesity classification faces similar challenges: BMI-for-age percentiles frequently misclassify muscular children, and growth charts fail to account for ethnic variations in body composition [16,17].

Advanced measures such as DEXA or MRI precisely assess fat quantity and location but remain costly and scarce in many settings [18]. Waist circumference can indicate visceral fat but suffers from inconsistent measurement protocols [19]. Relying on BMI alone also contributes to weight stigma, since about 30 percent of those classified as obese are metabolically healthy, while around 20 percent of people with normal BMI have high visceral fat and insulin resistance [20]. This has led experts to call for definitions that focus on metabolic health rather than weight alone.

## 4. Risk Factors for Obesity

**Figure 1:**
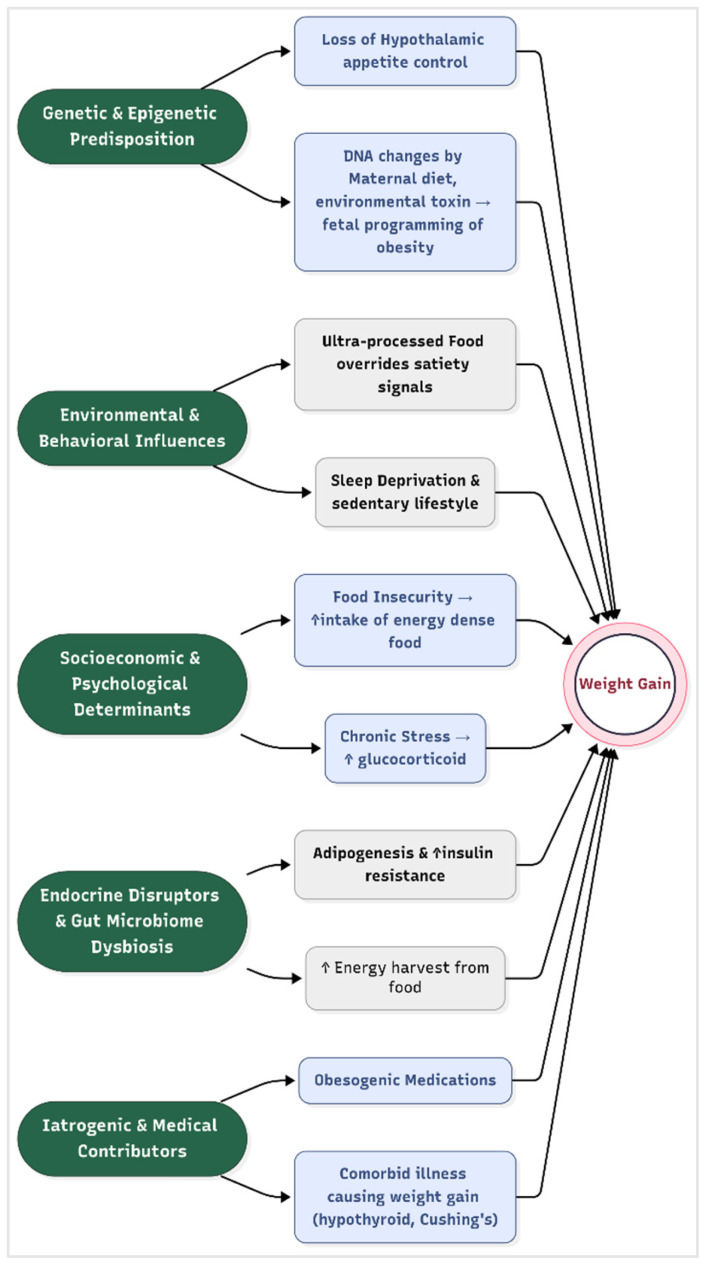
Risk factors for obesity.

Obesity is not merely a consequence of personal choice, but a multifactorial disease shaped by biological vulnerabilities interacting with environmental pressures. It arises from a complex interplay of genetic, environmental, behavioral, and socioeconomic factors that disrupt energy homeostasis, leading to sustained positive energy balance. While excessive caloric intake compared to expenditure remains the fundamental driver, emerging evidence highlights the nuanced biological and societal mechanisms that predispose individuals to weight gain (Figure 1).

- Genetic and Epigenetic Predisposition

Twin and family studies estimate that 40–70% of obesity risk is heritable, with over 1000 genetic loci implicated in weight regulation [21]. Key genes, such as the fat mass and obesity-associated gene (FTO), influence hypothalamic regulation of appetite and energy expenditure by modulating leptin and melanocortin signaling pathways [22]. Epigenetic modification, such as DNA methylation changes induced by maternal diet, stress, or environmental toxins, may also alter fetal programming of adiposity, creating intergenerational cycles of obesity [23].

Recent work on polygenic risk scores improved individual prediction of obesity susceptibility, revealing that high genetic risk can be attenuated by healthy lifestyle factors [24]. A recent study demonstrates how a composite exposome scores, including factors such as air pollution, diet, and sleep, correlate with metabolic biomarkers from childhood into adolescence, reinforcing the concept’s relevance to obesity development [25].

- Environmental and Behavioral Influences

The modern “obesogenic” environment promotes sedentary lifestyles and calorie-dense diets. Ultra-processed foods, which constitute > 60% of calories in high-income countries, are engineered to override satiety signals through high palatability and low fiber content [26]. Concurrently, urbanization reduced physical activity, with screen time displacing active pursuits. Sleep deprivation (<6 h/night) exacerbates metabolic dysregulation by elevating ghrelin (appetite-stimulating hormone) and reducing leptin (satiety hormone) levels [27]. Advanced geospatial analysis further supports this relationship; for example, deep learning models trained on satellite imagery have been able to predict obesity prevalence based on built environment characteristics with up to 90% accuracy [28]. Similarly, socioeconomic features of neighborhoods—such as poverty levels, rental housing proportion, and household structure—have been strongly linked to obesity rates through spatial machine learning approaches [29]. These findings highlight how behavioral patterns, such as sedentary lifestyles and poor dietary choices, are not merely personal decisions, but are significantly shaped by the surrounding physical and socioeconomic environment.

- Socioeconomic and Psychological Determinants

Lower socioeconomic status (SES) correlates strongly with obesity, particularly in high-income nations. Food insecurity paradoxically drives reliance on energy-dense, nutrient-poor foods due to cost constraints and limited access to fresh produce [30]. Psychological stressors, including chronic discrimination or depression, activate the hypothalamic–pituitary–adrenal (HPA) axis, promoting visceral fat deposition through glucocorticoid excess [31]. Additionally, neurobehavioral factors, such as reward-driven eating, are linked to dopaminergic signaling in the nucleus accumbens, increasing the susceptibility to overconsumption [32]. Emerging data on adverse childhood experiences reveal that early life trauma interacts with socioeconomic deprivation to epigenetically sensitize stress pathways, further elevating obesity risk [33].

- Endocrine Disruptors and Gut Microbiome Dysbiosis

Exposure to endocrine-disrupting chemicals (EDCs), such as bisphenol A (BPA) and phthalates, interferes with adipogenesis and insulin sensitivity by mimicking or blocking hormonal signaling [34]. Furthermore, gut microbiota composition, particularly reduced Bacteroidetes and elevated Firmicutes, enhances energy harvest from food and promotes systemic inflammation, as demonstrated in fecal transplant studies [35]. Recent metagenomic analyses identified specific bacterial strains whose loss predicts obesity development, opening the door to precision microbiome therapies [36].

- Iatrogenic and Medical Contributors

Certain medications, including antipsychotics (e.g., olanzapine), antidepressants, and glucocorticoids, promote weight gain through metabolic and appetitive effects. Weight gain is a well-established side effect of all anti-psychotics, especially the second-generation anti-psychotics such as clozapine and olanzapine, by antagonizing 5-hydroxy tryptamine receptor, dopamine receptor, and histamine and muscarinic receptors [37]. Medical conditions such as hypothyroidism, Cushing’s syndrome, and hypothalamic injuries also disrupt energy balance and cause weight gain [38].

## 5. Pathological Impact of Obesity on Human Health

**Figure 2:**
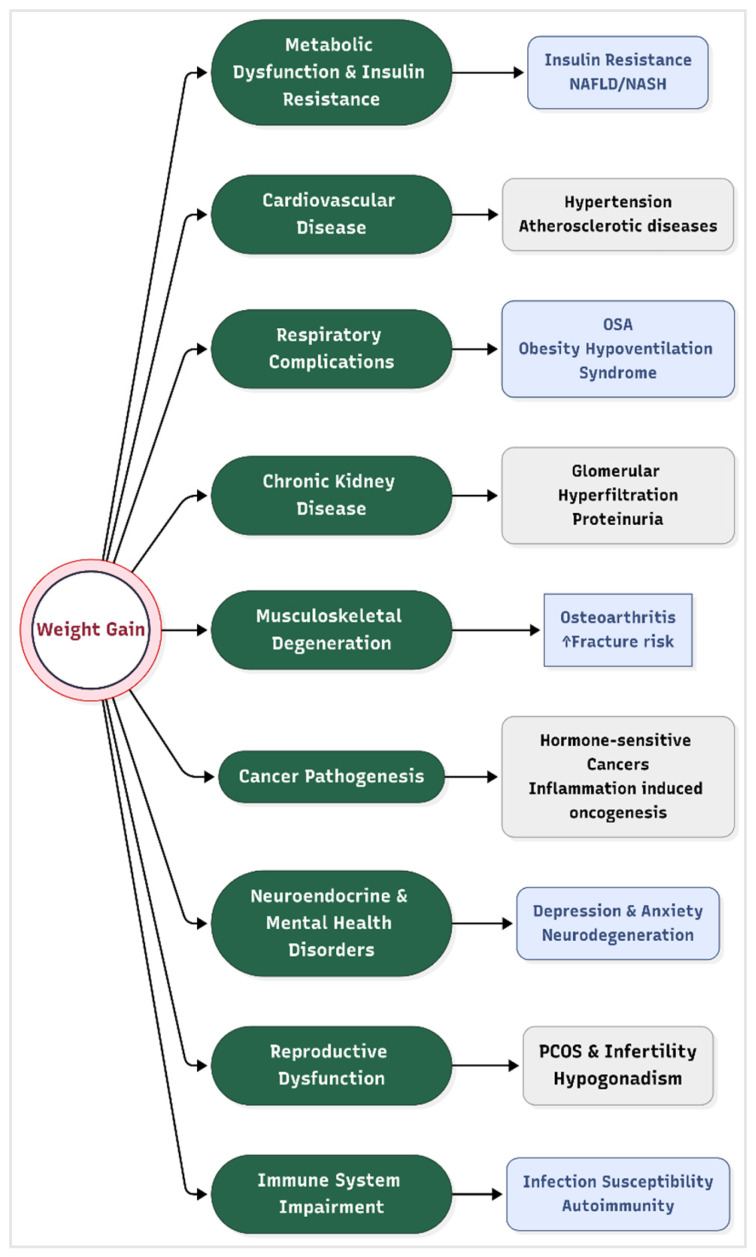
Complications of obesity. **Legend**: NASH = non-alcoholic steatohepatitis, NAFLD = non-alcoholic fatty liver disease, and PCOS = polycystic ovary syndrome.

Obesity is not merely a cosmetic concern, but a systemic disease that disrupts physiological equilibrium across multiple organ systems. It exerts profound effects on nearly every organ system that culminate in life-threatening comorbidities. These pathological processes are mediated by chronic low-grade inflammation, hormonal dysregulation, oxidative stress, and ectopic fat deposition, which collectively impair cellular and systemic homeostasis (Figure 2).

- Metabolic Dysfunction and Insulin Resistance

Excess adipose tissue, particularly visceral fat, secretes pro-inflammatory cytokines (e.g., TNF-α, IL-6) and free fatty acids that disrupt insulin signaling pathways. This leads to systemic insulin resistance, a hallmark of metabolic syndrome and type 2 diabetes (T2D) [39]. Hyperinsulinemia further exacerbates lipogenesis and inhibits lipolysis, creating a vicious cycle of fat accumulation and glucose intolerance. Non-alcoholic fatty liver disease (NAFLD), present in 70–90% of obese individuals, progresses to non-alcoholic steatohepatitis (NASH) and cirrhosis due to hepatic lipid overload and mitochondrial dysfunction [40].

- Cardiovascular Disease (CVD)

Obesity directly contributes to hypertension via increased blood volume, sympathetic nervous system activation, and endothelial dysfunction. Adipose-derived angiotensinogen promotes vasoconstriction and sodium retention, elevating blood pressure [41]. Additionally, dyslipidemia, which is characterized by elevated triglycerides, LDL cholesterol, and reduced HDL, accelerates atherosclerosis. Obese individuals exhibit a 2–3-fold higher risk of coronary artery disease, heart failure, and stroke due to chronic inflammation and plaque instability [42].

- Respiratory Complications

Central obesity mechanically compromises respiratory function by restricting diaphragmatic movement and reducing lung compliance. This causes predisposition to obstructive sleep apnea (OSA), where intermittent hypoxia triggers oxidative stress and systemic inflammation [43]. Obesity hypoventilation syndrome (OHS), characterized by chronic hypercapnia, further increases mortality risk due to right heart failure and pulmonary hypertension.

- Chronic kidney disease

Obesity is an independent risk factor for chronic kidney disease (CKD), accelerating glomerular hyperfiltration, proteinuria, and progressive renal decline; this risk is amplified by accompanying hypertension and diabetes [44]. Weight loss interventions have been shown to reduce albuminuria and slow CKD progression, underscoring the renal benefits of obesity management [45].

- Musculoskeletal Degeneration

Excessive mechanical loading on weight-bearing joints accelerates cartilage degradation, leading to osteoarthritis (OA). Adipokines such as leptin and adiponectin exacerbate joint inflammation and synovial fibrosis, independent of biomechanical stress [46]. Obesity also impairs fracture healing and increases osteoporosis risk due to chronic inflammation and vitamin D deficiency.

- Cancer Pathogenesis

Adipose tissue dysfunction contributes to cancer development through several interconnected biological mechanisms. One key factor is hyperinsulinemia and elevated levels of insulin-like growth factor 1 (IGF-1), which together stimulate the proliferation of tumor cells [47]. Additionally, chronic inflammation associated with obesity leads to increased production of reactive oxygen species (ROS), which can cause significant DNA damage and further promote oncogenesis. Another important mechanism involves the heightened synthesis of estrogen within adipose tissue, which plays a crucial role in the development of hormone-sensitive cancers such as breast and endometrial cancer. Epidemiological data highlight the magnitude of this risk, with obesity being implicated in approximately 40% of endometrial cancers and 20% of postmenopausal breast cancers [48].

- Neuroendocrine and Mental Health Disorders

Leptin resistance in the hypothalamus disrupts appetite regulation, perpetuating overeating [49]. Obesity is linked to a 30–50% higher risk of depression and anxiety, mediated by inflammation-driven alterations in serotonin and dopamine signaling [50]. Depression is more strongly linked to abdominal obesity than to overall obesity [51]. Neurodegenerative diseases, including Alzheimer’s, are exacerbated by obesity-induced blood–brain barrier disruption and amyloid-beta accumulation [52].

- Reproductive Dysfunction

In women, obesity induces hyperandrogenism and anovulation, contributing to polycystic ovary syndrome (PCOS) and infertility). In men, adipose tissue aromatase activity converts testosterone to estrogen, causing hypogonadism and reduced sperm quality [53]. Maternal obesity increases risks of gestational diabetes, preeclampsia, and congenital anomalies in offspring [54].

- Immune System Impairment

Obesity induces a state of chronic immune activation and dysfunction, impairing responses to pathogens while paradoxically increasing autoimmune disease risk. Adipose-resident macrophages shift to a pro-inflammatory M1 phenotype, secreting IL-1β and IL-6, which impair T-cell function and vaccine efficacy [55].

## 6. Historical Treatment Options for Obesity

Obesity management evolved significantly over the past century, with treatments ranging from early pharmacological interventions to invasive surgical procedures. Although many earlier approaches showed only modest effectiveness, their significant limitations, including serious side effects, high rates of relapse, and procedural risks, limited their long-term usefulness.

- Pharmacological Interventions
  ○ Amphetamines and stimulants (1930s–1970s): Amphetamines were first marketed in the 1930s as Benzedrine in an over-the-counter inhaler to treat nasal congestion. By 1937, amphetamines were available by prescription in tablet form and were used in the treatment of the sleeping disorder narcolepsy and ADHD. They were widely prescribed for weight loss in the mid-20th century due to their appetite-suppressing effects. However, their addictive potential, cardiovascular risks (e.g., hypertension, arrhythmias), and misuse led to strict regulation under the U.S. Controlled Substances Act in 1971 [56].
  ○ Fenfluramine/phentermine (1990s): This combination therapy enhanced serotonin release (fenfluramine) and norepinephrine reuptake inhibition (phentermine) to suppress appetite. While effective (average 10–15% weight loss), fenfluramine was withdrawn in 1997 after studies linked it to valvular heart disease and pulmonary hypertension [3].
  ○ Sibutramine (1997–2010): A serotonin–norepinephrine reuptake inhibitor, sibutramine reduced hunger and increased satiety. Despite 5–10% weight loss in trials, it was discontinued in 2010 after the SCOUT trial showed elevated cardiovascular events (e.g., stroke) in high-risk patients [4].
  ○ Orlistat (1999–present): A pancreatic lipase inhibitor, orlistat blocks dietary fat absorption, resulting in ~3–5% weight loss. It is still available, but its use is limited by gastrointestinal side effects (e.g., steatorrhea, fecal incontinence) and poor long-term adherence [57].
- Surgical Interventions
  ○ Jejunoileal bypass (1950s–1970s): This malabsorptive procedure involved bypassing most of the small intestine. While effective (~30% weight loss), it caused severe complications, including liver failure, renal stones, and malnutrition, leading to its abandonment [58].
  ○ Vertical banded gastroplasty (1980s): This restrictive surgery partitioned the stomach with staples and a band. Initial weight loss (20–25%) was often reversed due to staple-line breakdown and pouch dilation, with high reoperation rates [59].
  ○ Adjustable gastric banding (1990s–2010s): The Lap-Band^®^ restricted stomach capacity via an inflatable band. While safer than bypass surgeries, it resulted in only 15–20% weight loss, with frequent complications (band slippage, erosion) and a 40% long-term failure rate [60].
  ○ Roux-en-Y gastric bypass (RYGB, 1960s–present): Combining restriction and malabsorption, RYGB reduces stomach size and reroutes the small intestine. It remains the gold standard, with 25–30% sustained weight loss and remission of diabetes in 60–80% of patients. However, risks include dumping syndrome, micronutrient deficiencies, and rare but severe complications (e.g., anastomotic leaks) [5].

## 7. Emerging Medical Therapies for Obesity

**Figure 3:**
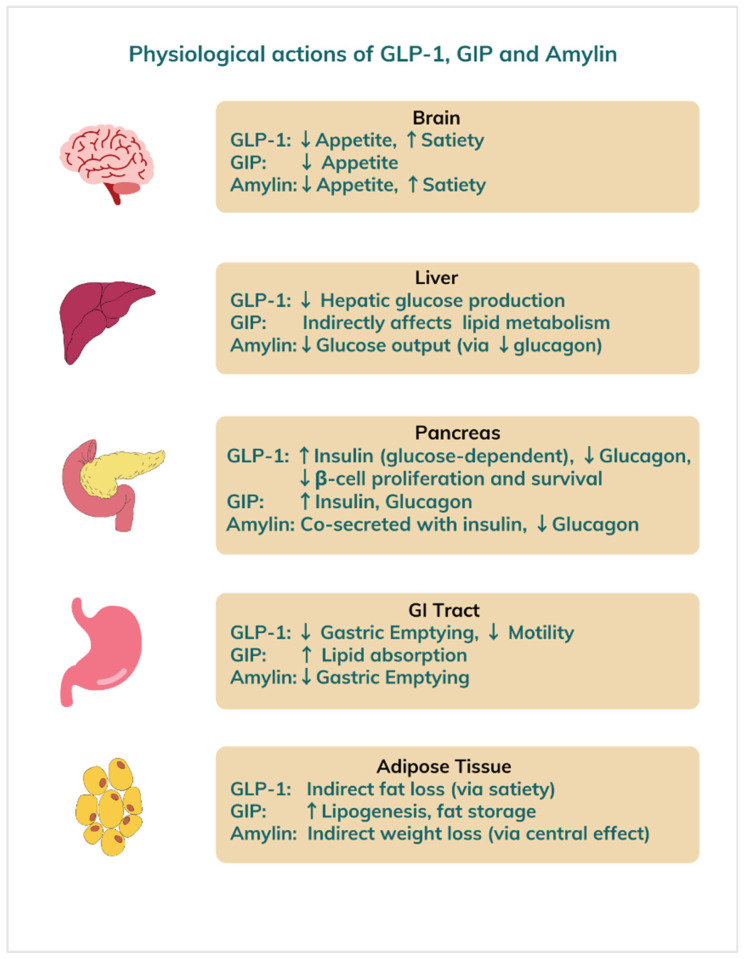
Physiological effects of GLP-1, GIP, and amylin hormones on different organs. **Legend:** GLP = glucagon-like peptide, GIP = glucose-dependent insulinotropic polypeptide.

Emerging medical therapies for obesity are rapidly evolving, offering new hope for individuals struggling with weight management (Table 1). Beyond traditional lifestyle interventions and older pharmacological agents, recent advances demonstrated significant weight loss in clinical trials by targeting appetite regulation and insulin sensitivity [6]. Most of these new medications work via glucagon-like peptide-1 (GLP-1), glucose-dependent insulinotropic polypeptide (GIP), and glucagon or amylin hormone receptors to exert their weight reducing effects (Figure 3).

GLP-1 and GIP are incretin hormones released by the gut after meals. They stimulate insulin release in response to rising blood glucose, suppress glucagon secretion, and slow gastric emptying to blunt postprandial spikes. Incretins also promote satiety, reducing food intake and making them prime targets in type 2 diabetes and obesity treatment [61].

Glucagon, secreted by pancreatic α-cells, raises blood sugar by stimulating hepatic glucose production when levels fall too low, thereby helping to maintain energy balance. Amylin, co-released with insulin from β-cells, likewise slows gastric emptying, suppresses glucagon, and enhances fullness to aid appetite control [62]. Novel drugs that mimic these hormone actions increase satiety, decrease intake, stabilize glycemia, boost energy expenditure, and promote weight loss.

### 7.1. Semaglutide

This is a long-acting glucagon-like peptide-1 (GLP-1) receptor agonist initially approved for type 2 diabetes under the trade names Ozempic^®^ (injectable, 2017) and Rybelsus^®^ (oral, 2019). In June 2021, the U.S. FDA approved a higher-dose injectable formulation (2.4 mg weekly) as Wegovy^®^ for chronic weight management in adults with obesity or overweight with at least one weight-related comorbidity. In STEP trials, the participants received counselling every 4 weeks, targeting a 500 kcal/day deficit and at least 150 min/week of moderate-intensity activity.

- Route of administration: It can be administered in both injectable (subcuteneous) and oral form as described above.
- Mechanism of action: Semaglutide mimics the effects of endogenous GLP-1, a gut-derived incretin hormone. It acts centrally on hypothalamic GLP-1 receptors to suppress appetite, enhance satiety, and reduce food intake. Peripherally, it slows gastric emptying and improves insulin secretion in a glucose-dependent manner, indirectly promoting weight loss [6].
- *Rybelsus* is an oral form of semaglutide, a modified GLP-1 analog in which the native peptide has been engineered for both enzymatic stability and albumin binding: specifically, an Aib (α-aminoisobutyric acid) substitution at position 8 to resist DPP-4 degradation and an attached C18 fatty diacid chain at lysine 26 (via a spacer) to promote reversible albumin binding and extend half-life [63]. Crucially, it is co-formulated with the small-molecule absorption enhancer SNAC (sodium N-[8-(2-hydroxybenzoyl)amino]caprylate), which transiently raises local gastric pH and facilitates transcellular uptake of semaglutide across the gastric epithelium [64]. Once absorbed, it mimics the similar action of endogenous GLP-1. For optimum absorption, Rybelsus should be taken first thing in the morning on an empty stomach, with no more than 4 ounces of water. At least 30 min should be allowed to pass before eating, drinking anything else, or taking other medications.
- Efficacy: In the semaglutide treatment effect in people (STEP) with obesity trials, semaglutide 2.4 mg weekly produced a mean placebo-adjusted weight loss of 14.9% over 68 weeks in STEP 1, and up to 16.0% in STEP 4 with continued use [6,8]. These outcomes are substantially superior to older therapies such as orlistat or phentermine/topiramate [65].
- Side effects: The most common adverse events are gastrointestinal, such as nausea (up to 44%), vomiting, diarrhea, and constipation. These effects are dose-dependent and occur primarily during dose escalation [65]. Rare but serious adverse events may include acute pancreatitis, gallbladder disease, and renal impairment. An FDA boxed warning exists for thyroid C-cell tumors based on rodent studies, contraindicating its use in patients with personal or family history of medullary thyroid carcinoma.

### 7.2. Tirzepatide

This is a first-in-class dual glucose-dependent insulinotropic polypeptide (GIP) and GLP-1 receptor agonist. It was first approved for type 2 diabetes (Mounjaro^®^) in 2022 and subsequently for obesity (Zepbound^®^) in 2023 for adults with BMI ≥ 30 kg/m^2^ or ≥27 with comorbidities. Most recently, Zepbound^®^ has been approved by the U.S. Food and Drug Administration in December 2024, as the first and only prescription medication indicated for moderate-to-severe obstructive sleep apnea (OSA) in adults with obesity [66].

- Route of administration: Tirzepatide is administered as a once-weekly subcutaneous injection.
- Mechanism of action: Tirzepatide is a 39–amino-acid peptide engineered as a dual agonist of the glucose-dependent insulinotropic polypeptide (GIP) and glucagon-like peptide-1 (GLP-1) receptors, combining both incretin activities within a single molecule to achieve prolonged plasma half-life [67]. By co-activating GIP and GLP-1 receptors on pancreatic β-cells, it amplifies cyclic AMP signaling and enhances glucose-dependent insulin secretion while concurrently suppressing glucagon release from α-cells, leading to improved glycemic control [68]. Activation of its GLP-1 component also delays gastric emptying, blunting postprandial glucose excursions and contributing to steadier overall glucose profiles [69]. In addition to these peripheral actions, its dual receptor engagement in hypothalamic feeding centers reduces appetite and caloric intake, resulting in substantial weight loss that surpasses effects seen with selective GLP-1 agonists [67].
- Efficacy: In the SURMOUNT-1 trial, tirzepatide 15 mg weekly achieved mean weight reductions of 20.9% over 72 weeks, with 50–57% of participants losing ≥20% of their body weight [7]. It also included regular lifestyle counselling sessions to reinforce a ~500 kcal/day deficit and ≥150 min/week of exercise during the trial. This surpasses all previously approved pharmacotherapies, including semaglutide.
- Side effects: Similar to GLP-1 agonists, it commonly causes gastrointestinal effects, including nausea (31%), diarrhea (22%), and constipation. Injection-site reactions and fatigue also occur. As with semaglutide, rare events include pancreatitis and gallbladder disease. There is a class effect boxed warning by the FDA for medullary thyroid carcinoma.

### 7.3. Retatrutide

This is currently under development. It is a triple agonist of GLP-1, GIP, and glucagon receptors. This triple receptor agonist has shown exceptional weight loss effects in early trials and is in phase 3 development as of 2025.

- Route of administration: It is administered as a weekly subcutaneous injection.
- Mechanism of action: Retatrutide combines appetite suppression (GLP-1, GIP) with increased energy expenditure via glucagon receptor stimulation. This multifaceted mechanism targets both caloric intake and energy metabolism and enhances insulin secretion, optimizes glucose homeostasis, and effectively regulates appetite [70].
- Efficacy: Findings from phase 1 to phase 3 clinical trials highlight retatrutide’s significant therapeutic efficacy, with marked reductions in body weight (up to 24.2% over 48 weeks) and improved glycemic control, underscoring its potential as a treatment for obesity and type 2 diabetes mellitus. In addition to its effects on weight and glucose regulation, retatrutide demonstrates potential benefits in reducing cardiovascular risk factors and managing non-alcoholic fatty liver disease, suggesting a broader role in the management of metabolic disorders [71].
- Side effects: Similar to other incretin-based agents, nausea, vomiting, and diarrhea are common. Glucagon activity may cause transient elevations in heart rate and hepatic transaminases.

### 7.4. Cagrilintide

This is a long-acting amylin analogue currently being investigated in combination with semaglutide (as CagriSema^®^) to enhance appetite suppression. In its phase 2 trial, participants in all arms followed a moderately hypocaloric diet (–500 kcal/day) and engaged in 150 min/week of moderate exercise, ensuring consistent lifestyle support alongside pharmacotherapy.

- Route of administration: It is administered as a weekly subcutaneous injection.
- Mechanism of action: Cagrilintide acts on amylin and calcitonin receptors within the area of the postrema and nucleus tractus solitarius, lowering food intake through both homeostatic and hedonic mechanisms. It slows gastric emptying, suppresses postprandial glucagon secretion, and reduces appetite via central hypothalamic pathways [63]. Its extended half-life enables convenient once-weekly dosing and a stable pharmacodynamic profile. When co-administered with the GLP-1 receptor agonist semaglutide (CagriSema^®^), they act synergistically to produce greater weight loss and improved glycemic control than either agent alone, without significantly increasing the incidence or severity of gastrointestinal adverse events [72,73]. In combination with GLP-1 receptor activation from semaglutide, this dual approach enhances anorectic effects.
- Efficacy: In a phase 2 study, the combination achieved mean body weight reductions of 15.6% at 32 weeks, compared to 5.1% with semaglutide alone [72].
- Side effects: The most common side effects of cagrilintide are gastrointestinal—mild-to-moderate nausea, vomiting, and constipation—which are generally transient and can be managed through gradual dose escalation and adequate hydration [74].

### 7.5. Orforglipron

This is a first-in-class, orally administered non-peptide GLP-1 receptor agonist under development for the treatment of obesity and type 2 diabetes. Unlike peptide-based GLP-1 receptor agonists such as semaglutide or liraglutide, orforglipron does not require injection, cold-chain storage, or co-formulation with absorption enhancers such as sodium N-[8-(2-hydroxybenzoyl)amino] caprylate (SNAC), making it a potentially more accessible and convenient option for patients [75].

- Route of administration: It is taken once a day as an oral pill.
- Mechanism of action: It binds to and activates the GLP-1 receptor to stimulate glucose-dependent insulin secretion, inhibit glucagon release, delay gastric emptying, and suppress appetite—mechanisms that contribute to improved glycemic control and substantial weight loss [76]. Preclinical studies demonstrated that orforglipron has a high oral bioavailability and favorable pharmacokinetics, supporting once-daily dosing [77].
- Efficacy: In a phase 2 clinical trial, orforglipron led to mean weight reductions of up to 12.6% over 36 weeks in people with obesity, a result comparable to injectable GLP-1 analogues [76].
- Side effects: Common side effects included nausea, vomiting, and diarrhea, which were dose-dependent and tended to decline over time. Importantly, no cases of severe hypoglycemia were reported, supporting its favorable safety profile when used without concomitant insulin or sulfonylureas [77].

### 7.6. Setmelanotide

Setmelanotide (Imcivree^®^) was approved in 2020 for obesity due to proopiomelanocortin (POMC), leptin receptor (LEPR), or PCSK1 deficiency, and later for Bardet–Biedl syndrome [78].

- Route of administration: It is administered as a weekly subcutaneous injection.
- Mechanism of action: It is a melanocortin-4 receptor (MC4R) agonist that restores signaling disrupted in genetic obesity disorders. Unlike GLP-1 therapies, setmelanotide acts directly on hypothalamic satiety pathways.
- Efficacy: In trials, 80% of patients with POMC deficiency and 45% with LEPR deficiency achieved ≥10% weight loss over one year. Hunger scores also improved dramatically.
- Side effects: Injection site reactions, hyperpigmentation, and nausea are common. Skin darkening occurred in ~60% of trial participants. No serious long-term safety concerns have been observed [78].

### 7.7. Tesofensine

Tesofensine is a novel triple monoamine reuptake inhibitor originally developed for neurodegenerative diseases but repurposed for obesity after marked weight loss was observed in early studies [79]. It is currently being evaluated in phase III clinical trials for chronic weight management.

- Route of administration: It is administered as a once-daily oral capsule.
- Mechanism of action: Tesofensine inhibits the presynaptic reuptake of dopamine, norepinephrine, and serotonin in appetite-regulating centers of the brain, thereby suppressing hunger and enhancing satiety [79].
- Efficacy: In the pivotal phase IIb “TIPO-1” trial, obese patients treated with 1.0 mg of tesofensine daily for 24 weeks experienced a mean weight loss of 12.8 kg (≈11% of body weight), compared with a 2.2 kg loss in the placebo group.
- Side effects: The most commonly reported adverse events included dry mouth, nausea, insomnia, headache, diarrhea, and constipation. Dose-dependent increases in heart rate (up to 8 bpm) and blood pressure (1–3 mmHg) were noted, with an overall withdrawal rate of 13% versus 6% for placebo.

### 7.8. Bimagrumab

Bimagrumab is a human monoclonal antibody targeting activin type II receptors, initially investigated for muscle-wasting syndromes, but now explored for obesity and metabolic health. It has completed phase IIa studies and is currently in phase IIb development in combination trials [80]. In the bimagrumab trial, participants received standardized diet and exercise guidance, targeting a 500 kcal/day deficit and 150 min/week of moderate-intensity activity throughout the 48-week study, ensuring the muscle-preserving effects of the myostatin inhibitor were assessed against a consistent lifestyle backdrop.

- Route of administration: It is administered as a monthly intravenous injection.
- Mechanism of action: Bimagrumab is a fully human monoclonal antibody that exerts its anabolic effects on skeletal muscle by dual blockade of activin type II receptors, thereby neutralizing multiple negative regulators of muscle growth [81,82]. It acts via several pathways as follows:
  ○ High-affinity binding to ActRIIA and ActRIIB: Bimagrumab binds both activin receptor type IIA (ActRIIA) and type IIB (ActRIIB) with high affinity, preventing endogenous ligands—principally myostatin (GDF-8), activin A, activin B, and growth differentiation factor 11 (GDF-11)—from engaging the receptor complex.
  ○ Inhibition of Smad2/3 signaling: Ligand-activated ActRII receptors normally recruit and phosphorylate Smad2/3 transcription factors, which then translocate to the nucleus to upregulate atrophy-associated genes (e.g., atrogin-1, MuRF-1) and suppress protein synthesis pathways. Bimagrumab’s receptor blockade abolishes Smad2/3 phosphorylation, shifting the balance toward muscle protein accretion.
  ○ Promotion of myoblast differentiation and hypertrophy: By neutralizing multiple TGF-β family ligands simultaneously, bimagrumab not only counteracts myostatin’s anti-anabolic signal, but also blocks activin-mediated inhibition of myogenic differentiation. The result is enhanced myoblast fusion, increased fiber cross-sectional area, and a more than two-fold greater hypertrophic response than myostatin inhibition alone.
  ○ Reversible endocrine modulation: Activin signaling in the anterior pituitary regulates follicle-stimulating hormone (FSH) secretion. In healthy adults, bimagrumab transiently suppresses FSH and subtly alters luteinizing hormone (LH) responses without impacting downstream sex steroid levels; these effects fully reverse after drug clearance.
  ○ Net clinical effect: Through these combined actions—pan ligand blockade at ActRIIA/B, inhibition of catabolic Smad signaling, and potentiation of anabolic myogenic pathways—bimagrumab consistently increases lean body mass, strength, and functional outcomes in sarcopenic and muscle-wasting populations. By blocking activin IIA and IIB receptors, bimagrumab inhibits myostatin and related ligands, promoting skeletal muscle hypertrophy and enhancing adipose tissue loss through increased energy expenditure.
- **Efficacy:** In a 48-week phase II randomized trial of adults with type 2 diabetes and obesity, intravenous bimagrumab (10 mg/kg every 4 weeks) led to a 20.5% reduction in fat mass and a 3.6% increase in lean mass versus placebo, with an overall weight loss of 6.5% compared to 0.8% for placebo.
- **Side effects:** Bimagrumab was generally well-tolerated; reported adverse events included transient gastrointestinal symptoms, muscle cramps, and mild elevations in liver enzymes. No serious cardiovascular signals were observed.

## 8. Limitations of New Obesity Pharmacotherapy

Obesity is a chronic, relapsing condition that pharmacotherapy alone cannot “cure.” Semaglutide and other incretin-based therapies produce unprecedented weight losses (15–25%). Bimagrumab achieved less weight loss but was able to increase the lean mass along with loss of adipose tissue. It should be noted that diet and exercise counseling was an integral part of many of these new mediation trials. By embedding these novel medications within robust diet and exercise programs, these trials isolate the pharmacologic effect while modeling the comprehensive, multi-modal approach needed for maximal and sustained weight loss. In real-world practice, achieving similar outcomes will require maintaining that same level of lifestyle support alongside long-term drug therapy. Thus, discontinuation of medication may lead to substantial regain of weight when adequate healthy diet and exercise routines are no longer followed. Semaglutide users can regain two-thirds of their loss within one year off treatment [6]. Similar rebound effects are reported for other agents, such as tirzepatide [7]. Biological counter-regulatory mechanisms, such as reductions in leptin, rises in ghrelin, and adaptive metabolic slowing, cause increased hunger and energy conservation after weight loss [83]. Behavioral inertia also plays a role: without sustained lifestyle changes, appetite-suppression wanes and old dietary patterns return [84]. Effective and lasting obesity management requires a focus on fundamental lifestyle strategies, including consistent caloric restriction through whole foods, avoidance of ultra-processed products, regular physical activity, and behavioral support, to maintain a negative energy balance and prevent weight regain [85,86].

## 9. New Therapeutic Approaches on the Horizon

Beyond hormone-based drugs and lifestyle interventions, several cutting-edge strategies are emerging that target the underlying molecular and microbial drivers of obesity. These investigational approaches hold promise for more durable and personalized treatment.

### 9.1. Epigenetic Modulation

Obesity is associated with abnormal epigenetic marks, such as DNA methylation changes and altered histone acetylation, that perpetuate dysfunctional fat cells and energy imbalance. Experimental therapies aim to reset these marks:

- DNA methyltransferase inhibitors, such as low-dose 5-azacytidine, improved lipid oxidation and insulin sensitivity in animal models by demethylating promoters of metabolism related genes [87].
- Histone deacetylase modulators, including selective HDAC3 inhibitors, increase expression of genes involved in mitochondrial growth and activation of brown fat, leading to higher resting energy use [88].
- Natural products such as resveratrol, curcumin, and omega 3 fatty acids are under study for their capacity to induce beneficial chromatin changes in liver and fat tissue, with early data showing modest improvements in body composition [89].

### 9.2. Fecal Microbiota Transplantation

The gut microbiome influences metabolism, appetite, and energy absorption. Transplanting stool from lean donors seeks to correct microbial imbalance in people with obesity:

- Single-dose transplantation has temporarily improved insulin sensitivity and reduced liver fat, though sustained weight loss has been small without repeat procedures [90].
- Combining transplantation with low fermentable fiber intake enhances donor microbe growth, supports production of beneficial fatty acids, and yields larger reductions in abdominal fat over six months [91].
- Future studies use defined bacterial mixtures rather than whole stool to create standardized, safe, and scalable therapies aimed at specific metabolic pathways.

### 9.3. Noncoding RNA-Based Therapeutics

- MicroRNA inhibitors (antagomirs) targeting miR-103 and miR-107 improve insulin sensitivity and reduce adiposity in diet-induced obese mice by enhancing hepatic and peripheral insulin signaling [92].
- Conversely, miR-196a mimics promote the browning of white adipose tissue—upregulating uncoupling protein 1 and other thermogenic genes—thereby increasing energy expenditure and conferring resistance to diet-induced obesity [93].
- Long noncoding RNA modulation—for example, lipid nanoparticle-mediated silencing of the adipocyte-specific lncRNA lncOb—restores leptin expression, reduces fat mass, and improves glucose tolerance in obese mouse models [94].

These cutting-edge approaches such as editing epigenetic marks, engineering the gut microbiome, and using RNA-based therapies could work alongside or even outperform current treatments and bring us closer to personalized obesity care.

## 10. Long Term Strategies for Sustained Weight Loss

- Calorie Restriction and Dietary Quality

Long-term weight loss requires a sustained calorie deficit, but calorie quality is equally important for adherence, satiety, and metabolic health [95]. Network meta-analyses show that continuous restriction, alternate-day fasting, and time-restricted eating each yield comparable weight loss and maintenance [96].

Higher-protein diets (≥25 percent of energy) enhance fullness, preserve lean mass, and boost resting energy expenditure versus standard protein levels [97]. Dietary fiber from whole grains, legumes, fruits, and vegetables further slows gastric emptying and regulates gut hormones to support blood sugar control and satiety [98].

Ultra-processed food intake correlates with greater weight gain and obesity, independent of macronutrients [85]. Replacing these with minimally processed items, such as fresh produce, lean proteins, and nuts, improves diet quality and aids long-term management [99].

The Mediterranean diet, rich in olive oil, vegetables, legumes, nuts, and fish, promotes weight maintenance and cardiovascular health even without strict calorie limits [100]. Mindful eating practices, such as watching portions, avoiding distractions, and not eating late at night, further enhance energy regulation.

- Physical Activity

Regular physical activity is vital for initial weight loss and even more important for preventing weight regain. The United States Physical Activity Guidelines recommend 200 to 300 min per week of moderate intensity aerobic activity or 75 to 150 min per week of vigorous aerobic activity, plus muscle strengthening exercises on at least two days each week [101].

Aerobic exercise boosts total energy expenditure, increases fat oxidation, and supports heart health. Resistance training preserves lean body mass and slows the drop in resting metabolic rate that often follows weight loss [102].

Non-exercise activity thermogenesis (NEAT), which refers to the energy expended during everyday movements such as walking, fidgeting, and household tasks, can account for a difference in energy expenditure of up to 2000 kilocalories per day. Factors such as occupation, leisure activities, genetics, and even food intake can influence NEAT levels, making it a crucial factor in maintaining long-term weight stability [103].

Combining aerobic and resistance exercises yields the best results for body composition and weight stability. Meta analyses show that this combined approach reduces weight regain by two to three kilograms over twelve months compared to aerobic activity alone [104]. Interrupting prolonged periods of sitting with brief bouts of activity each hour also improves metabolic flexibility [105].

- Behavioral and Supportive Interventions

Behavioral strategies lay the groundwork for lasting changes in diet and activity. Self-monitoring via daily weighing and keeping food and exercise logs predicts long-term success. Individuals who engage in regular self-monitoring tend to lose and maintain up to three kilograms more than those who do not [106].

Ongoing support through monthly or bimonthly telephone sessions or in-person coaching reduces weight regain by about 2.5 kg over one year compared to minimal follow up [86]. Motivational interviewing uses patient-centered communication to enhance intrinsic motivation and adherence and yields more sustained weight loss than standard education [107].

- Sleep and Stress Management

Chronic sleep deprivation, defined as less than seven hours per night, disrupts appetite regulation by increasing ghrelin and decreasing leptin, leading to greater caloric intake and weight gain [108]. Meta-analyses indicate that improving sleep duration and quality reduces obesity risk and supports long-term weight maintenance [109]. Psychosocial stress promotes emotional eating, particularly of high-calorie, palatable foods, and contributes to central fat accumulation through elevated cortisol levels [110]. Stress management techniques, including mindfulness-based stress reduction and cognitive behavioral therapy, have been shown to lower emotional eating and reduce weight regain by up to 1.5 kg over one year [111].

## 11. Future Direction in Obesity Treatment Research

With the rapid expansion of anti-obesity pharmacotherapy beyond GLP-1 receptor agonists, future directions are poised to harness multi-agonist peptides, novel hormone pathways, and precision medicine approaches. Dual GIP/GLP-1 agonists such as tirzepatide have already demonstrated unprecedented mean weight losses of >20% in the SURMOUNT-1 trial, validating co-agonism as a superior strategy [6]. Building on this, triple-receptor agonists—targeting GLP-1, GIP, and glucagon receptors—are in phase 2 development and promise further synergistic gains by combining appetite suppression, increased energy expenditure, and enhanced lipid metabolism [112]. Parallel efforts with long-acting amylin analogues, exemplified by cagrilintide, are exploring combined regimens to modulate both homeostatic and hedonic feeding circuits more robustly than single-pathway agents [74]. Beyond peptide hormones, emerging small-molecule melanocortin-4 receptor agonists aim to fine-tune central satiety pathways with oral dosing, while microbiome-targeted therapies and gene-editing technologies hold long-term promise for individualized metabolic resets. Together, these innovations signal a shift toward personalized combination regimens that not only reduce weight more effectively, but also address heterogeneity in patient response, comorbidity profiles, and tolerability.

## 12. Conclusions

Obesity is a chronic and relapsing disease in which recent pharmacologic advances, including semaglutide, tirzepatide, and emerging tri agonist therapies, have produced substantial short-term weight loss. However, discontinuation of these medications often leads to significant weight regain due to persistent biological drivers such as hormonal adaptations, decreased metabolic rate, and the return of previous eating and activity patterns. While pharmacotherapy can initiate meaningful weight reduction, it does not fully address the underlying dysregulation of energy balance.

To maintain weight loss after medication, patients must rely on consistent lifestyle strategies. These include a diet focused on minimally processed whole foods, adequate intake of protein, fiber, fruits, and vegetables, and a sustained calorie deficit. Regular physical activity, combining both aerobic and resistance exercises, is essential to preserve lean mass and support metabolic health. In addition, behavioral support strategies, such as self-monitoring, structured counseling, and accountability systems, are critical for long term success. Adequate sleep and effective stress management further contribute to a sustainable approach.

Looking ahead, the development of new pharmacologic agents targeting appetite, energy expenditure, and fat metabolism offers promise for enhancing long-term outcomes. When combined with evidence-based lifestyle interventions, these therapies may significantly improve the durability of weight loss and provide patients with more effective tools in the management of obesity.

## Figures and Tables

**Table 1:** Comparison of modern anti-obesity medication.

Drug (Brand Name)	Indication of Use	Mechanism of Action	Side Effects	Route of Administration
Semaglutide (Wegovy^®^)	Chronic weight management in adults with obesity or overweight with at least one weight-related condition	GLP-1 receptor agonist	Nausea, vomiting, diarrhea, constipation, abdominal pain	Subcutaneous injection
Semaglutide (Rybelsus^®^)	Type 2 diabetes; used off-label for weight loss	GLP-1 receptor agonist	Nausea, diarrhea, decreased appetite	Oral
Tirzepatide (Mounjaro^®^, Zepbound^®^)	Zepbound: obesity, OSA; Mounjaro: type 2 diabetes	Dual GIP and GLP-1 receptor agonist	Nausea, diarrhea, vomiting, constipation	Subcutaneous injection
Ritatrutide	Investigational for obesity and type 2 diabetes	Triple agonist: GIP, GLP-1, and glucagon receptors	Nausea, vomiting, diarrhea	Subcutaneous injection
cagrilintide + Semaglutide (CagriSema^®^)	Investigational for obesity and type 2 diabetes	Combination of cagrilintide (amylin analog) and semaglutide (GLP-1 agonist)	Nausea, vomiting, decreased appetite	Subcutaneous injection
Orforglipron	Investigational for obesity and type 2 diabetes	Non-peptide oral GLP-1 receptor agonist	Nausea, vomiting, diarrhea	Oral
Setmelanotide (Imcivree^®^)	Chronic weight management in patients with rare genetic obesity disorders (e.g., POMC deficiency)	MC4 receptor agonist	Skin hyperpigmentation, nausea, injection site reactions	Subcutaneous injection
Tesofensine	Investigational for obesity	Triple Monoamine reuptake inhibitor (dopamine, norepinephrine, serotonin)	Dry mouth, nausea, constipation, increased heart rate	Oral
Bimagrumab	Investigational for obesity and sarcopenia	human monoclonal antibody targeting Activin type II receptor (ActRII); increases muscle mass, reduces fat	Muscle spasms, diarrhea, fatigue	Intravenous infusion

**Legend:** GLP: glucagon-like peptide, GIP: glucose-dependent insulinotropic polypeptide, OSA: obstructive sleep apnea, and POMC: proopiomelanocortin.
